# A Systematic Review of Imaging Techniques for the Botanical and Geographical Classification of Coffee

**DOI:** 10.3390/foods15050821

**Published:** 2026-03-01

**Authors:** Leticia Tessaro, Yhan da Silva Mutz, Davide Orsolini, Rosalba Calvini, Natália de Oliveira Souza, Giulia Mitestainer Silva, Alessandro Ulrici, Cleiton Antônio Nunes

**Affiliations:** 1 Department of Chemistry, Federal University of Lavras, Lavras P.O. Box 3037, Minas Gerais, Brazil; 2 Department of Food Science, Federal University of Lavras, Lavras P.O. Box 3037, Minas Gerais, Brazil; 3 Department of Life Sciences, University of Modena and Reggio Emilia, Pad. Besta, Via Amendola, 2, 42122 Reggio Emilia, Italy; davide.orsolini@unimore.it (D.O.); rosalba.calvini@unimore.it (R.C.); alessandro.ulrici@unimore.it (A.U.)

**Keywords:** coffee, authentication, digital imaging, HSI, MSI, cultivar, variety, species, discrimination, machine learning, neural networks

## Abstract

With evolving consumption trends, the coffee market is experiencing increasing demand for high-quality, traceable coffees, which, in turn, has led to price growth. Therefore, due to its increased economic value, coffee has become a constant target of fraudulent actions. As result, many analytical techniques have been explored as tools for coffee classification and authentication, of which the use of digital, hyperspectral and/or multispectral imaging is noteworthy. This type of analysis provides rapid, non-destructive, environmentally friendly, and increasingly accessible alternatives to conventional analytical methods. By consulting three different databases, this work systematically revised articles published in the last 10 years, which utilize digital image analysis and hyper/multispectral imaging for the botanical and geographical classification and authentication of coffees. The reviewed studies (*n* = 17) demonstrate that, when paired with classification algorithms, discrimination across species, origins, and quality categories can be achieved. A critical point to highlight is the importance of using whole beans and standardizes roast degree to avoid biasing the models. Concerning digital images, relying solely on color features limits the robustness of the classification models. Incorporating complementary textural and shape features is thus necessary to capture the coffee botanical or geographic information, as shown in a minor number of the selected studies. In a similar fashion, for hyper/multispectral imaging, there is still potential to further exploit the spatial information, thus achieving the technique’s full potential. The evidence indicates that image-based methods are steadily progressing into reliable tools for coffee authentication.

## 1. Introduction

Coffee is among the most traded commodities worldwide and the second most popular beverage. According to the United States Department of Agriculture (USDA) Foreign Agricultural Service, global coffee production for the 2024/2025 season was estimated at approximately 175 million 60-Kg bags [1]. The demand for high-quality coffee has increased substantially, driven by its distinctive sensory attributes, which have generated highly interested consumers [2]. The coffee is considered specialty grade when it scores above 80 under the cupping standards established by the Specialty Coffee Association (SCA). These coffee market prices may reach values ten times those of conventional commodity coffees [3]. Among the high-priced coffee class are the products originating from regions with geographical indications (GI), which are also associated with qualities and reputations that arise essentially or exclusively from their geographical environment [4]. Nevertheless, this added value makes these coffees particularly vulnerable to fraudulent practices and counterfeiting [5].

The chemical composition of coffee beans, as well as their physical properties (size, color, density, and defects), are influenced by genetic and environmental factors, including botanical species, altitude, humidity, temperature, and management practices [6]. This variability gives the product a wide range of aromas, flavors, and qualities, but it also represents an analytical challenge, especially in authenticity control, variety differentiation, and geographical origin certification [5].

Traditionally, the evaluation and classification of coffees have been carried out through visual inspection and sensory analysis, based on the experience of specialized tasters. However, these methods are inherently subjective, susceptible to fatigue and the individual perception of the evaluator, which compromises the consistency and reproducibility of the results [7]. On the other hand, conventional laboratory techniques such as chromatography and mass spectrometry, although accurate, are expensive, destructive, require sample preparation, and generate chemical waste [8]. In this context, it becomes necessary to adopt rapid, sustainable, and reproducible analytical strategies that provide physicochemical and morphological information about coffee without compromising its integrity.

The development of image-based technologies has improved food characterization and analysis, and coffee is one of the products that has benefited most from these innovations. Techniques such as digital imaging, multispectral imaging (MSI), and hyperspectral imaging (HSI) allow for the simultaneous extraction of spatial and spectral information from samples, providing a “fingerprint” associated with the chemical composition, color, and texture of the grain [9,10,11]. Digital image analysis, for example, uses the red, green, and blue channels of the RGB space to reproduce variations in color and brightness, generating quantitative data that can be processed by chemometric algorithms [12]. MSI and HSI techniques integrate spectroscopy and computer vision, capturing images in multiple bands of the electromagnetic spectrum and allowing the evaluation of physicochemical characteristics in a simultaneous, fast, and non-invasive way [9,13,14].

The combination of these approaches with multivariate and machine learning models has enabled pattern recognition for the automated identification of species (*Coffea arabica* and *Coffea canephora*), control of roasting level, and authentication of geographic origin [7,15,16,17]. These methods overcome human limitations, offering fast, objective, reproducible, and non-destructive analyses.

In addition to their analytical performance, these methodologies are fully aligned with the principles of green analytical chemistry (GAC), which seeks to reconcile technological innovation, efficiency, and sustainability. GAC proposes reducing energy and reagent consumption, eliminating waste, and using non-destructive and in situ techniques, avoiding sample preparation and disposal steps [18,19]. Digital and spectral imaging techniques fulfill these principles by eliminating the need for solvents and chemical steps, reducing costs and analysis time, and allowing the use of portable equipment, which favors direct and sustainable analysis of coffee authenticity.

Furthermore, thanks to their rapid, non-destructive, and information-rich analytical capabilities, digital imaging, MSI, and HSI can be easily integrated into automated sorting lines, enabling real-time quality assessment during industrial processing or quality control.

**Figure 1:**
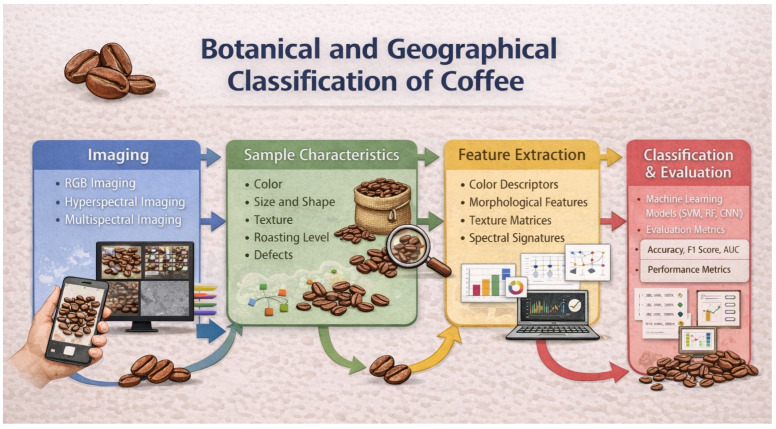
Schematic diagram summarizing the key themes considered in the studies on the botanical and geographical classification of coffee using images.

Based on these considerations, this systematic review sheds light on articles published in the last 10 years that use digital images or multi/hyperspectral images in the botanical and geographic classification of coffee. From this bibliographic dataset, the current state-of-the-art on this topic is examined, providing a critical assessment of their achieved outcomes and full potential of the imaging techniques considered, along with their limitations and future prospects. To facilitate the integration and visualization of the main themes discussed in this review, Figure 1 provides a schematic diagram summarizing the key aspects considered in the studies on the botanical and geographical classification of coffee using digital, hyperspectral and multispectral imaging.

## 2. Systematic Methodology

This review was guided by the Preferred Reporting Items for Systematic Reviews and Meta-Analyses (PRISMA) guidelines in order to identify, select, appraise, and synthesize the studies [20]. A PRISMA checklist is provided in the Supplementary Material (File S1).

### 2.1. Information Sources and Selection Criteria

This systematic review was conducted on September 10, 2025, through research in the three databases: Web of Science, Scopus, and the International System for Agricultural Science and Technology (AGRIS). The study was conducted using the search components SC1 (Coffee), SC2 (hyperspectral OR multispectral OR RGB), and SC3 (imag*). This review considered original research articles published over the last 10 years (2015–2025) and excluded revisions and theses. The first-step screening was conducted by reading the titles, keywords, and abstracts. Articles were removed in this initial screening if the study did not use digital image, multispectral, or hyperspectral data for the authentication or discrimination of species, varieties, or geographical indications of coffee. Articles not written in the English language, without a DOI, and that have not taken their own images for the dataset were also excluded. This systematic review was not registered under PRISMA.

### 2.2. Risk of Bias Assessment

To minimize the risk of bias, during the eligibility assessment, studies were required to report (i) a validation strategy (e.g., cross-validation, external validation, independent test set), (ii) data preprocessing procedures, and (iii) a clear description of the chemometric method applied. Articles lacking these details were excluded. Two reviewers independently evaluated risk of bias and methodological quality, resolving disagreements by consensus with a third reviewer.

### 2.3. Focus Questions

The focus questions agreed with the problem, intervention, comparison, and outcome method by PICO (Problem, Intervention, Comparison, and Outcome). The research questions were:

**Q1.**  What has been achieved with the use of digital images in the authentication and discrimination of species, varieties, and geographical indications in coffee?
**Q2.**  What extracted features contributed more to the authentication and discrimination of species, varieties, and geographical indications in coffee?
**Q3.**  What chemometric approaches are used to authenticate coffee from digital image data?
**Q4.**  What has been achieved with the use of hyperspectral and multispectral images in the authentication and discrimination of species, varieties, and geographical indications in coffee?
**Q5.**  How has the spatial information of hyperspectral and multispectral data been applied to the authentication and discrimination of species, varieties, and geographical indications in coffee?
**Q6.**  What are the chemometric approaches used to authenticate and discriminate coffee from the hyper/multispectral data?

## 3. Results

### 3.1. Search Results and Frequency of Research Publications

**Figure 2:**
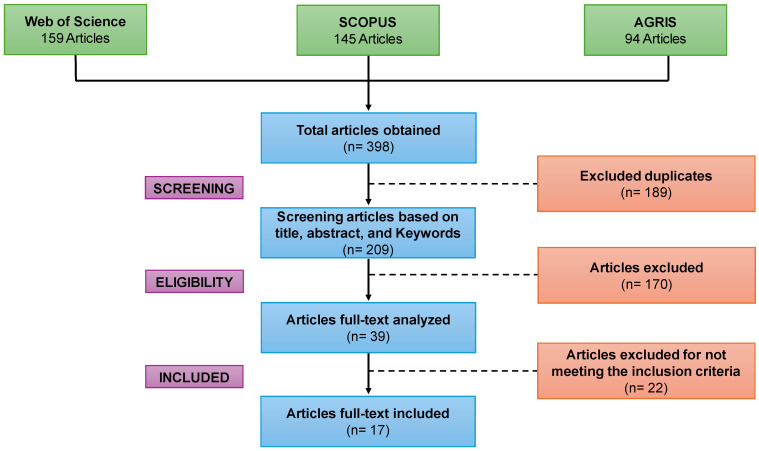
The schematic diagram for the selection of articles included based on the PRISMA methodology.

Data extraction and results assessment were performed independently by two reviewers. The results of the systematic search are presented in the PRISMA flow chart (Figure 2). A total of 398 studies from the Scopus, Web of Science, and AGRIS databases were selected. Lastly, a total of 17 studies were selected for quantitative analysis. All useful information obtained from each study was extracted and compiled in Tables, as presented in the following sections.

**Figure 3:**
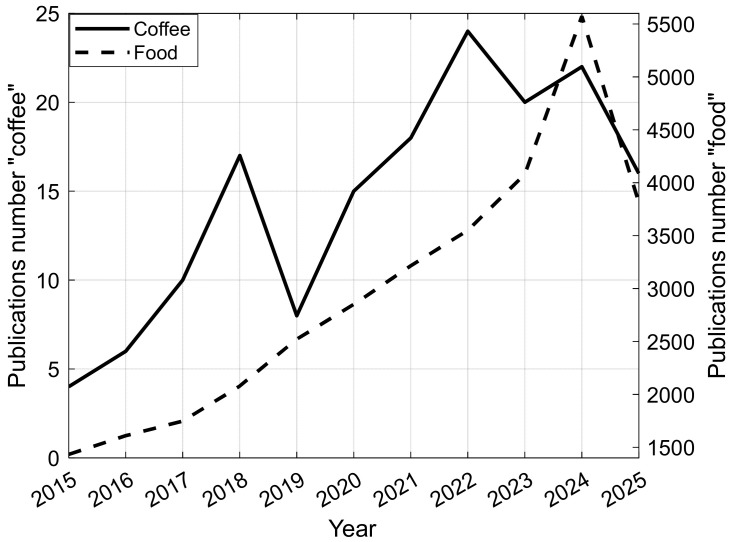
Number of publications over the last 10 years about the use of images or multispectral/hyperspectral images and food, specifically coffee, in the Web of Science database. Search date 27 November 2025.

In Figure 3, the number of publications related to the use of images or multispectral/hyperspectral data in the broad area of food, specifically coffee, over the last 10 years is shown. The growing number of publications year by year demonstrates that these approaches are efficient for various tasks in food science.

The data demonstrate the relevance of this systematic review, shedding light on knowledge about all recent studies published related to the use of images or multispectral/hyperspectral images to authenticate or discriminate varieties, species, and geographic indications of coffee.

### 3.2. Digital Imaging in Species, Variety, or Geographic Indication Authentication

Digital imaging is a non-destructive technique that simulates human visual perception to acquire, process, and analyze images for decision-making [21]. Essentially, a digital image is a numerical representation of a two-dimensional object, composed of a matrix of discrete image elements known as pixels. Each pixel carries specific information about light intensity and color, typically stored in three channels in the visible range (red, green, and blue—RGB) [22]. The operational principle involves illuminating a sample, capturing the reflected light using sensors, and subsequently processing the data to extract features such as color, shape, size, and texture [23].

The development of computer vision happened in the 1960s, with early academic projects attempting to teach computers to recognize simple objects. Although initial applications were confined to the medical and industrial fields, the technology began to permeate the agricultural sector in the late 1980s and 1990s as processing power increased, offering an automated alternative to the tedious and subjective manual inspection of crops [24].

In the food industry, digital imaging has become a standard tool for quality control and safety assessment, and it is widely used for fruits and vegetables, where color, shape, and texture are key indicators of ripeness, freshness, and integrity. Several studies employ RGB images to classify ripening stages, identify mechanical injuries, or detect post-harvest deterioration [21,22,23,25,26]. In cereals and grains, the technique also yields consistent results for evaluating characteristics such as size, uniformity, blemishes, surface defects, and degree of processing, often using RGB histograms, texture metrics, or contour analysis [27].

For the coffee sector, the use of digital image tools has emerged as a robust, fast, and low-cost alternative. Conventional inspection methods, such as the manual classification of coffee beans, not only hinder trade processes but also impose challenges on workers, such as fatigue and stress, potentially impacting the quality of coffee beans [28]. The analyzed works demonstrate that computer vision can be applied in various situations, such as in the authentication of geographical origin [29], in examining the characteristics of bean particles during grinding [30], and in evaluating roasting levels [31].

**Figure 4:**
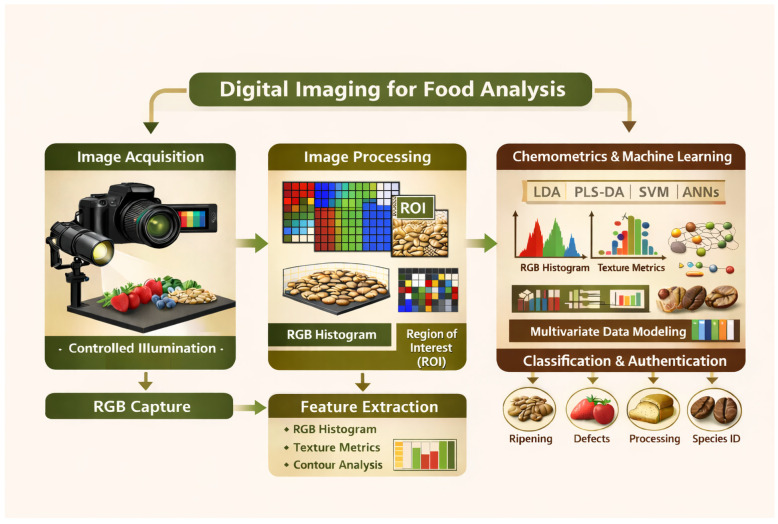
Overview of digital imaging in food analysis, illustrating the steps of acquisition, processing, feature extraction, and chemometric tools.

The following sections, based on the current literature derived from the selected articles in this systematic review, focus on the authentication or classification of coffee based on species, variety, or geographical origin through the application of digital imaging. In further detail, Section 3.2.1 describes the characteristics of the samples analyzed in the selected works. Section 3.2.2 focuses on the technical aspects of image acquisition, data preprocessing, and features extracted. Section 3.2.3 examines the multivariate data analysis approaches and deep learning methods used, along with the results obtained. The overall workflow of digital imaging applied to food analysis, including image acquisition, processing, feature extraction, and multivariate data modeling for classification and authentication, is illustrated in Figure 4.

#### 3.2.1. Sample Characteristics for Coffee Authentication Using Digital Imaging

Digital image acquisition can be performed using simple devices, such as smartphones, webcams, digital cameras, and scanners, which record color information. However, digital images contain not only color information but also morphological and textural properties. Based on these considerations, digital imaging allows for characterization of coffee samples based on features that are somehow linked to color, morphology, and texture, while a more comprehensive chemical characterization is only possible with multi/hyperspectral imaging [32]. Therefore, in digital RGB imaging, the disposition of the coffee beans, and particularly their physical form and condition, are crucial factors for achieving good results. Table 1 shows the physical and biological parameters of the samples used in the selected works.

The bean’s physical form provides unique characteristics that can generate a fingerprint, enabling the classification of species, varieties, and geographical indications. For example, green coffee beans of *Coffea arabica* and *Coffea canephora* exhibit distinct physical characteristics, such as size, shape, and color, that make them easily distinguishable [37]. Waliyansyah & Hasbullah (2021) [7] compared classification methods for digital image processing of Indonesian green coffee beans from *Coffea arabica* and *Coffea canephora*. The images were captured using a mobile camera, and the classification models yielded satisfactory results.

Also, the differentiation of varieties (or cultivars) within a species can be achieved through unique color and shape traits, and this can be done manually or mechanically. In this context, digital imaging can be used to automate and objectively differentiate coffee cultivars. Alhasson & Alharbi (2025) [33] developed a smartphone application that leverages squeeze vision transformer technology for classifying the varieties Harari, Khawlani, Nabari, Laqamti, and Briah of *Coffea arabica*. For this purpose, digital images of whole beans were acquired for both green and roasted coffee. The performance metrics demonstrate the model’s capability to predict coffee type, thereby understanding the differences between beans.

Lastly, the task of differentiating geographic indications in coffees of the same species and variety is analytically more complex. In this case, it is not possible to distinguish coffee samples by their geographical origin using visual assessment. According to the literature, a range of elementary analyses is described for this task, such as IRMS (isotope ratio mass spectrometry), FAAS (flame atomic absorption spectrometry), ETAAS (electrothermal atomic absorption spectrometry), and ICPAES (inductively coupled plasma atomic emission spectrometry) [38]. No article selected in this systematic review aimed at classifying the geographic origin of a singular variety of coffee using digital images. However, two recent works have demonstrated the differentiation of geographical indications between different species or varieties using digital images. Baqueta et al. (2024) used roasted and ground coffee samples to authenticate the Amazonian Robusta cultivars of *C. canephora* from Rondônia (Brazil) against pure *C. canephora* and adulterated *C. arabica* samples [17]. The images were obtained using a smartphone and utilized in a classification model based on DD-SIMCA (Data Driven Soft Independent Modeling of Class Analogy) [17]. In another study, Candeias et al. (2025) [16] authenticated an unspecified variety of *C. canephora* from South Bahia against the same species of coffee from Espirito Santo, Brazil. The images were obtained by a scanner. The DD-SIMCA method was used to authenticate the South Bahia region, resulting in 100% sensitivity, specificity, and accuracy against other coffees.

Exclusively, these works, Baqueta et al. (2024) [17] and Candeias et al. (2025) [16], were unique in utilizing ground coffee; the other works included in the review used whole bean coffee samples. This is a concern when using digital images for authentication, as physical visual characteristics such as size, shape, and morphology of the beans are lost after grinding.

Furthermore, when dealing with roasted coffee, it is important to verify that color differences resulting from different roasting levels do not interfere or overlap with the information of interest. The study published by Mendonça et al. (2009) demonstrated that, after grinding, no significant differences in chroma values could be observed between *C. arabica* and *C. canephora* samples [39]. Chroma is a color feature that represents color saturation and tone and was measured with a tristimulus colorimeter. However, the difference between these species was observed using color feature luminosity [39]. This evidence shows that results from digital images (RGB, grayscale, etc.) of ground coffee beans should be carefully evaluated, along with their features, to ensure that chemometric tools do not model the degree of roasting rather than the characteristics of the coffee beans.

Indeed, another relevant factor to consider when using digital images is the degree of roasting. During roasting, the coffee bean undergoes significant color changes; initially green, the bean presents a yellow-green coloration during the process. After complete roasting, the beans present a reddish-brown coloration due to the formation of melanoidins resulting from the Maillard reaction. In addition to the Maillard reaction, other physicochemical changes occur during roasting, such as Strecker degradation, caramelization of sugars, modification of chlorogenic acids, and alteration of proteins and polysaccharides [40,41]. As a result, roasting is a challenging process to replicate.

The color obtained after roasting the beans is of utmost importance for sorting the product into three levels of roasting: light, medium, or dark [40]. A widely applied roasting evaluation method is the Agtron scale, developed by the Specialty Coffee Association of America (SCAA) and Agtron Inc. The measurement is made with specific equipment, an Agtron spectrophotometer, which returns an Agtron value, a scale from 25 to 95, with lower values indicating darker roasts and higher values indicating lighter roasts [42]. After the roasting process, several differences other than the color in the bean’s characteristics to consider are: length, width, thickness, individual volume, apparent volume, apparent density, and porosity [43]. Furthermore, dark roasts tend to reduce pore uniformity and increase bean fragility, leading to a less uniform grind distribution. On the other hand, light roasts result in a more consistent grind size distribution, as pore sizes remain more uniform [42].

A significant distinction among the coffee beans’ roasting degrees lies in their metabolomic profiles. The literature describes how the roasting process causes the gradual degradation of chlorogenic acids and other phenolic compounds, associated with a decrease in antioxidant activity. Meanwhile, other antioxidant compounds are formed, such as those derived from the Maillard reaction [40,44]. However, this myriad of chemical distinctions is not directly perceivable by digital image analysis. Therefore, it is not possible to quantify the extent to which different roasting processes impact the coffee chemistry.

One factor that deserves highlighting when working with roasted coffee beans is the difficulty in replicating the roasting process due to its complexity. This step should be standardized for digital image analysis to ensure that the modeling focuses on the coffee’s characteristics and not just the roast level. Studies generally lack standardization of coffee colors, with no reported information on standardization via color analysis, Agtron, spectrophotometry, or evaluation by a roast specialist. For example, the study of Septiarini et al. (2024) clearly shows four different roasting degrees between authenticated varieties [15]. Although only shape features were used to model the results, as already detailed, the degree of roasting influences the size and porosity of the coffee bean. Therefore, differences in roasting levels directly influence the shapes of the beans.

For other studies that use color features without a method to standardize a similar roasting level for the entire sample set, the concern may be even greater, as these studies may be neglecting a source of variation in their data, the color, and instead presenting results without specifying the possible biases and limitations.

#### 3.2.2. Color and Shape Features Extraction

In digital image analysis, color features are typically extracted from RGB images [45]. Besides the color, shape features can be extracted from an object in a digital image; in this case, the object is a coffee bean. Shape features include area, perimeter, circularity, length, width, and others. As distinct sample information is carried in these different characteristics, exploring all that can be extracted from digital images is a way to ensure better, more reliable results.

**Figure 5:**
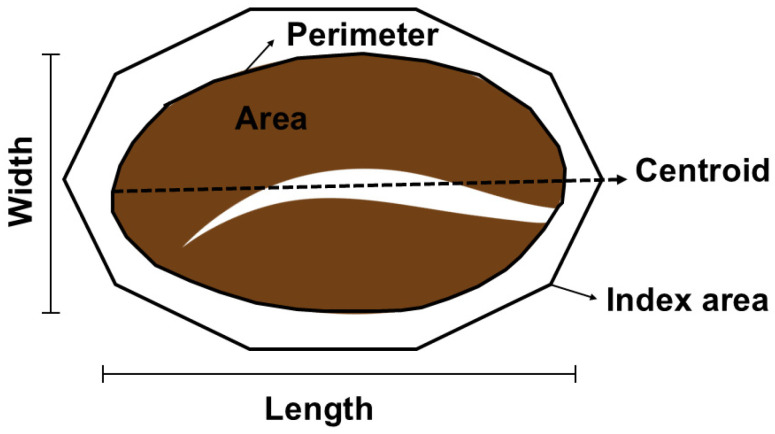
Shape features that can be extracted from a digital image of a coffee bean.

Figure 5 illustrates a schematic representation of the global shape features that can be extracted from a coffee bean. From these, other derived features can be calculated. The shape features focus on geometric descriptors based on contours or edges that capture facial deformation and curvature [46]. These parameters can be acquired manually or automatically by algorithms.

Table 2 shows an overview of parameters and features extracted from digital images of coffee samples in the studies reported in this review.

The morphology of a coffee bean shows shape features that are important and differ between species and varieties in terms of area, equivalent diameter, perimeter, and percentage of roundness [44]. In the selected works, one extracted only shape features, four extracted only color features, and three extracted both color and shape features.

Waliyansyah & Hasbullah (2021) extracted only color features from the coffee digital images to convert into texture features [7]. For this, a 3.2 megapixel sensor was employed with a sample distance of 20 cm, and the image was cropped to a 1000 × 1000 pixel resolution. The RGB images were pre-processed into grayscale [7]. Using the Gray Level Co-occurrence Matrix (GLCM) method, the following texture parameters were identified for analysis: energy, correlation, homogeneity, and contrast. The parameters dataset was modeled using machine learning algorithms, achieving great performance metrics in cross-validation [7].

The works that utilized only RGB are Baqueta et al. (2024) [17] and Candeias et al. (2025) [16]. Baqueta et al. (2024) provide the roasting protocol for all coffees and report that the adulterants were roasted under conditions similar to those of the authentic coffees to achieve comparable final colors [17]. In the context of fraud detection, this specific lack of color evaluation is less problematic, as real-world adulteration practices would likewise aim to mimic the color of genuine products, and a color-based model would remain effective. For geographical-origin authentication, however, the absence of a quantified roast-level comparison between authentic samples and coffees from other regions may introduce bias, as part of the model’s variance could reflect differences in roasting rather than true geographical variation. The authors do not discuss this possibility or provide evidence supporting their claim that a color-based method could, independent of the roasting process, lead to a separation of geographical origin.

To improve the generalizability of color-based authentication, future studies should consider evaluating samples across multiple roast levels, ideally using standardized measurements such as an Agtron scale or instrument-based colorimetry. In the study by Candeias et al. (2025), the reliance on commercially available samples further limits control over roast-level variation [16]. Although the authors conduct an exploratory analysis, noting tonal differences among coffees from distinct origins, they do not substantiate whether and why these differences may arise from geographical factors or if they instead reflect divergent roasting styles, extraction conditions, or freeze-drying procedures. Although this does not detract from the validity of their findings, as in the study discussed in the previous paragraph, it underscores the need for careful qualification of claims regarding the authentication of geographical information, which may be valid only under the evaluated process conditions.

The only work that utilized solely shape features was Septiarini and collaborators (2024) [15]. The acquisition was made by placing a single coffee bean in a minibox studio with an LED lamp, and capturing the images with a 12 MP smartphone camera positioned 30 cm away from the studio at a 30-degree angle from the beans. Initially, the original images were resized from 6000 × 3376 to 640 × 480 pixels. The ROI was selected in the digital images and pre-processed using gray scaling and histogram equalization. After this, 14 shape features were extracted. The evaluation of parameter significance was performed using the gain rate (GR) attribute, which assesses the limitation of information gain on each parameter in the model, that is, whether all extracted features are significant. Only eight features showed GR values greater than 0: aspect ratio, eccentricity, equivalent diameter, area, length, perimeter, width, and complexity. From these features, seven datasets were generated to determine the most discriminating combination of shape features for distinguishing four coffee bean species (Arabica, Robusta, Liberica, and Excelsa). Several machine learning methods were employed, where the Decision Tree model showed the greatest performance indexes in a model that utilized only four parameters: aspect ratio, eccentricity, equivalent diameter, area, and length [15].

This review highlights the importance of carefully selecting feature extraction techniques tailored to the specific characteristics of the images and the analysis objectives. The integration of such techniques can provide a more comprehensive and reliable representation of the images, thereby enhancing the accuracy and effectiveness of image-based methods. For instance, Widyaningtyas et al. (2025) evaluated 90 textural and 11 color features for discriminating palm civet coffee artificially adulterated with regular *C. arabica* [36]. To handle that amount of data, the author assessed several optimization algorithms (which led to the selection of the best subset of features) alongside different modelling approaches. Evaluating different optimization approaches for the random forest classifier, the authors observed accuracies of 0.962 and 0.981 for models using 51 and 5 features, respectively. More than the efficacy of different optimization algorithms, these results demonstrate the importance of understanding the extracted features and the potential of combining texture and color [36].

In contrast to the manual, user-based, feature extraction, the works published by Alhasson & Alharbi [33], Cuaya-Simbro and collaborators [34], and Molla & Mitiku [35] utilized convolutional neural networks (CNNs), which automatically extract features from digital images. CNNs are composed of several layers, which have convolutional filters capable of detecting patterns; the first layers can detect simple features (color gradients and basic textures); the intermediate layers learn to combine patterns (contour and geometric shape); and finally, the deepest layers can extract abstract shapes [47]. However, what is extracted are mathematical representations that indirectly encode this information. CNN features are not interpretable as direct geometric measurements; therefore, it is not possible to determine whether the CNN extracted specific shapes, such as diameter, edge, or area [48]. CNN is a deep learning model that performs end-to-end learning, i.e., it learns how to map and select significant features directly from the raw inputs (images) towards the defined output (classes), without requiring user-based decision making [48]. In summary, the images of coffee beans were introduced into CNN, which performed automatic extraction, and the shape and color features used in these works cannot be specified. Therefore, these works are not relevant to the discussion of this section as they do not fall within the scope, as it is not possible to identify the extracted features.

Overall, the reviewed studies indicate that manually engineered features are typically limited to either color-based or shape-based descriptors, rarely integrating both features simultaneously. This restriction may reduce the descriptive power of traditional feature extraction approaches. In contrast, the strong performance observed in convolutional neural networks may be partially attributed to their ability to exploit the full spatial and color information contained in the image, capturing complex and complementary patterns that manual methods overlook. This work highlights that excellent and robust results can be achieved when exploring the full potential of information that can be obtained from images.

#### 3.2.3. Classification Approaches and Metrics for Evaluating and Validation

Table 3 presents the details of the model building and classification procedures used in the selected works.

##### Sampling of the Reviewed Studies

Multivariate classification methods are widely applied in coffee-related research across several analytical platforms, including infrared spectroscopy, nuclear magnetic resonance, and chromatography [49,50,51]. Recently, the use of digital image-based analysis for such tasks has also been gaining adoption, as shown in Table 4. The basic principle behind multivariate classification is pattern recognition, either for class assignment (class modelling approaches) or constructing discrimination boundaries between predefined classes (discrimination techniques), where each approach relies on its own set of model-specific calculations [52]. However, before discussing model metrics, algorithmic comparisons, or optimization strategies, it is essential to examine the sampling process.

Sampling is the foundation of any scientific experiment because the selected subset of individuals (the sample set) is expected to represent the population’s physical and chemical characteristics. All natural materials, including fruits and biological matrices, are heterogeneous at scales relevant to sampling [53]. Consequently, the sampling protocol must address this inherent heterogeneity to ensure that population variance is adequately captured. Therefore, the sample size is critical, as small datasets can be biased towards a specific characteristic or may be insufficient to approximate an accurate population profile [54]. Larger datasets enable statistically sound inferences, including the application of the central limit theorem when sample sizes are sufficiently large [55]. However, in a practical sense, sample availability can be a limiting factor in the field of coffee and food science, making a case-by-case assessment necessary.

Of the eight studies presented in Table 4, only two reported the number of coffee samples analyzed, while the remaining only disclosed the number of images used. Although the number of images is relevant as the analytical input for the models, it must be distinguished from the sample count. Having multiple images from a single sample constitutes an analytical replicate and accounts for the variability of the analytical instrument (i.e., the camera) in the analysis. In contrast, robust classification or authentication models require biological variance inherited from botanical or geographical factors, which can be achieved only through multiple distinct biological samples [53]. Thus, a high number of images alone does not guarantee that the underlying biological variability of coffee is adequately captured.

Alhasson & Alharbi used a SqueezeNet-based CNN with a Vision Transformer enhancement to classify five Saudi coffee types at four roasting levels [33]. The authors report acquiring 12,310 images, each depicting a single bean; however, it remains unclear how many batches or harvests were used, and therefore, the extent of biological variation represented by each coffee type. The authors also note the use of two different lighting conditions to enhance image diversity, implying that the number of unique beans may be half the total number of images. In another work, Molla & Mitiku (2025) employed a CNN combined with a histogram of oriented gradients (HOG) to extract deep features of coffee beans and classify between five varieties of Ethiopian coffee using a radial basis function (RBF) SVM [35]. The authors specify using 1200 images, of which half were generated by augmenting the original dataset of 600 images. No specifications regarding batches or harvests were provided for the coffee samples, thereby limiting the interpretation of the extent of sample representation for each of the five varieties.

Beyond the general lack of information regarding sample counts or diversity across most studies (Table 4), those employing CNNs also artificially increased their image dataset through data augmentation. Data augmentation expands the number of images by applying controlled transformations and is regarded as a strategy for reducing model overfitting in deep learning models [56,57]. The classical augmentation techniques, such as rotations, flips, and crops, help the model to become more robust to lesser distortions or differences in image acquisition, thereby improving generalization within the same dataset domain [57]. However, these approaches should be employed with caution and a full understanding of their limitations. Although the increased number of images may give the sense of a more comprehensive and robust dataset, data augmentation does not introduce new biological variability, which only arises from the sampling process through the adoption of different batches and harvests. Consequently, a model trained on a large number of augmented but biologically redundant images may result in a narrow data representation, limiting its ability to generalize for the purposes intended, such as to classify entire cultivars or geographic origins [58,59].

##### Model Performances

Considering the sampling description constraints outlined in the previous section, the following discussion evaluates the reported model performances. Particular attention is given to how sampling representativeness, or the lack thereof, affects model performance, interpretability, and the confidence with which the findings can be generalized.

In the scope of coffee classification using multivariate methods, authentication tasks are among the most approached [60]. Preventing counterfeiting and fraudulent practices is essential, as coffee prices vary significantly based on its farming and post-harvest processing, as well as the final product’s sensory attributes. Although fraud can be relatively easily perpetrated, for example, by simply blending different coffees, differentiating an authentic product can pose a significant analytical challenge [61]. This difficulty is specifically pronounced when the coffee is already roasted and ground, in which the visual cues that normally aid in distinguishing species, varieties, or strange bodies are largely lost [62].

Baqueta et al. (2024) investigated the geographical-origin authentication of indigenous *C. canephora* produced in the Amazon region of Brazil, against *C. canephora* from Espírito Santo, and artificially adulterated coffee samples [17]. The authors extracted RGB histograms from digital images of 100 indigenous canephora coffee samples to build a DD-SIMCA model for the target class. The model achieved a 98% sensitivity for the 100 indigenous coffees. DD-SIMCA is a class modeling approach that can be applied as a one-class classifier (OCC). It models the target class by capturing within-class similarities and defining the class boundaries using the score distance (SD) and the orthogonal distance (OD) from a PCA of the target samples [54]. In validation, the authors evaluated 100 artificially adulterated samples, of which only five were misclassified as pure indigenous coffee. Seven different adulterants were roasted according to the indigenous coffee roasting procedure and blended in proportions ranging from 10% to 70% (w/w). Additional validation against *C. canephora* from other producing regions also resulted in only five misclassifications out of 100. These results demonstrate the effectiveness of DD-SIMCA in modelling class structure and highlight its potential as a powerful tool for authentication.

The use of OCC methods is widely regarded as the current standard for developing authentication models [63]. OCC methods focus exclusively on modelling the target class. Thus, with appropriate sampling and sufficient representation of class variance, they can successfully characterize the authentic class and reject samples from unknown or external classes [64]. Consequently, such models are well-suited for detecting both known and unknown fraud types, especially DD-SIMCA, which makes no a priori assumptions about data distribution, thereby yielding a data-driven, optimized model. This strategy contrasts with discriminant classification approaches, which rely on identifying differences among classes within the dataset (i.e., authentic and not authentic samples), and therefore lack the “soft” classification capability that allows a model to abstain from forcing a sample into a predefined class [54,63,64]. However, discriminant approaches are beneficial when dealing with overlapping classes, as simply modeling within-class variation is insufficient to achieve satisfactory results, and explicit class separation must be enforced.

In another authentication study, Candeias et al. (2025) aimed to authenticate the geographical origin of instant *C. canephora* samples from Southern Bahia, Brazil [16]. The authors used the isolated and combined RGB, HSV, and grayscale histograms of 30 roasted ground instant coffees from Southern Bahia as input to build the authentic class model with DD-SIMCA. In the test set, all the DD-SIMCA models achieved 100% sensitivity except for the grayscale model, which reached 80%. Regarding specificity, the model using all three color histograms as well as the HSV-only model perfectly assigned all 60 external samples, whereas the RGB and Grayscale models achieved 93.3% and 81.7%, respectively. The external set included 30 *C. canephora* samples from other producing regions, 15 *C. arabica* samples, and 15 samples of unknown botanical and geographical origins. These results provide further evidence of the effectiveness of digital colorimetry combined with DD-SIMCA for coffee authentication. The authors also investigated the use of histograms to discriminate coffees from Espirito Santo from those of an undeclared origin using PLS-DA. In this case, the HSV and combined histograms models achieved 100% accuracy, whereas the isolated RGB and Grayscale models achieved 95% and 75% respectively. Because the degree of botanical and geographical differences between the groups was not known, interpreting the source of discrimination, whether chemical, visual, or both, is challenging. Nonetheless, the results underscore the strong potential of color attributes for both authentication and discrimination of coffees.

The studies by Baqueta et al. (2024) [17] and Candeias et al. (2025) [16] present robust results, supported by well-designed sampling strategies and external validation, which offer strong evidence for the reliability of the findings. However, as discussed in Section 3.2.2, a critical topic remains unaddressed in both: the explicit measurement of color or roast level in the final samples.

Apart from the two previously discussed studies employing OCC approaches, none of the remaining works provided explicit information on the number of samples or on the sampling procedures used. Without these details, it is not possible to determine whether the biological variability of the systems under study was adequately represented. Consequently, the reported performance of the models should be interpreted with caution, as it primarily reflects behavior within the specific datasets analyzed, limiting the extent to which the findings can be generalized to broader biological contexts.

Shifting from OCC, the six other works summarized in Table 4 all adopt supervised discriminant models, which divide the variable space into a number of regions corresponding to the number of considered classes by modeling the differences among them [65]. Among these, four studies rely on traditional machine-learning classifiers rather than CNN.

Molla & Mitiku (2025) sought to classify five different varieties of Ethiopian coffee using image features derived from a hybrid CNN–HOG descriptor as input for an RBF-SVM model [35]. The authors report an overall test-set accuracy of 97.5%, demonstrating the strong discriminative potential of the CNN–HOG descriptors and the classification capability of RBF-SVMs. However, the absence of details regarding hyperparameter tuning for the RBF-SVM, as well as the lack of any form of cross-validation, raises concerns about potential overestimation of model performance. Without specification of critical parameters such as C and γ, the model cannot be reproduced, and its generalizability remains uncertain.

Widyaningtyas et al. (2025) investigated the authentication and purity assessment of civet coffee with varying proportions of arabica coffee (0–100%) using color- and texture-based features [36]. They extracted 101 features (color and texture) and employed a wrapper-based feature selection framework using KNN, SVM, and Random Forest classifiers, optimized with several metaheuristic algorithms. Their best model was the Random Forest optimized with the Grey Wolf Optimizer, achieving 98.1% accuracy. While SVM and KNN yielded lower accuracies, the authors do not discuss the causes of this performance gap. Furthermore, the superior performance of Random Forest may reflect the complementary nature of the selected descriptors, which combine color and shape-based features, as previously discussed, potentially enhancing class separability due to their distinct and synergistic discriminatory capacities. Although no cross-validation scheme was reported, the authors explicitly state that evaluating model generalization remains an area for future development, which is an appropriate acknowledgment of the model’s current limitations.

By observing Table 4, the majority of studies adopted a discriminant approach, and the models achieving the highest classification performance were all non-linear. These findings align with the general properties of real-world image data, which often contain hierarchical, high-dimensional patterns across texture, shape, and color [66]. Linear methods may thus be insufficient to fully capture the complexity of such distributions. Given these limitations, CNN emerges as a particularly promising approach for coffee image classification. With end-to-end approaches, CNN models and the numerous features that can be learned indeed increase the capability of image-related analysis n [67,68,69]. Of the studies summarized in Table 4, two employed end-to-end CNN, but only one study presented an external validation approach.

Alhasson & Alharbi (2025) developed and validated a deep learning model to classify five Saudi coffee varieties across four roasting levels using a hybrid architecture combining SqueezeNet with a Vision Transformer enhancement [33]. The model was trained on 12,310 images and achieved an overall test set classification accuracy of 78%. However, because information on the biological variance of each coffee type is not provided, the generalizability of these findings may be limited. Nevertheless, the use of multiple roast levels likely directs the model to learn roast-independent features that discriminate among the coffee types, suggesting that CNNs are capable of capturing refined discriminatory patterns even across complex species-related tasks.

Overall, a clear preference for one-class classifiers is observed in authentication tasks, whereas non-linear discriminant models and convolutional neural networks are preferred in multi-class coffee classification problems. Indeed, OCC approaches are favored for characterizing authentic products and detecting unknown forms of adulteration, whereas discriminant methods are more suitable for explicitly separating predefined classes. Among discriminant models, CNN-based architectures are particularly advantageous for their ability to learn hierarchical, high-dimensional representations directly from image data, integrating color, texture, and structural information in an end-to-end framework. These characteristics make CNNs particularly suitable for complex classification scenarios involving multiple varieties, origins, and processing conditions. However, at present, the effectiveness of this class of techniques cannot be fully established, as it remains contingent on rigorous sampling design, explicit biological replication, and comprehensive external validation to ensure generalizability beyond the training domain.

### 3.3. Multispectral and Hyperspectral Imaging in Authentication of Species, Variety or Geographic Indication

While standard digital images are limited to a maximum of three channels in the visible range, MSI and HSI systems allow the acquisition of substantially larger amount of spectral information, capturing data across several wavelengths and considering spectral regions also outside the visible range.

**Figure 6:**
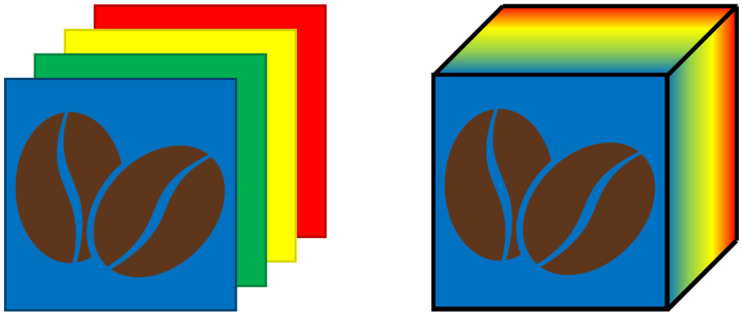
Graphical representation of a multispectral image (**left**) and a hyperspectral image (**right**).

The main distinction between the two techniques is that MSI records a set of individual images at specific, discrete wavebands, while HSI collects data over a continuous spectral range. Consequently, a multispectral image is composed of as many single-channel images as the number of acquired wavebands, whereas a hyperspectral image forms a continuous three-dimensional data cube, the so-called hypercube, in which every pixel contains a full spectrum [70]. This distinction is shown in Figure 6.

MSI and HSI combine aspects of conventional imaging and spectroscopic techniques, integrating spatial and spectral information to assess physical and chemical properties of a sample in a rapid, non-destructive way [71].

Both MSI and HSI were first employed in the late 1970s and the beginning of the 1980s for remote sensing applications, i.e., to examine the planet’s surface and geochemical properties using sensors mounted on aircraft or satellites [72,73]. In the following decades, these systems were adapted to a more controlled environment, leading to their application in fields such as the pharmaceutical and medical research [74,75], forensics [76,77], preservation of cultural heritage [78,79], waste sorting [80,81], and many others. MSI and HSI are particularly valuable in food analysis, as they allow the assessment of the spatial distribution of sample chemical constituents while simultaneously visualizing morphological and textural features. In scientific literature, many examples can be found of the application of both techniques to the safety and quality assessment and to the authentication of food products such as meat and seafood [82,83], cereals [84,85], fruits and vegetables [86,87], herbs and spices [88,89], as well as liquid products [90].

MSI and HSI have also been widely used to analyze coffee, both at pre- and post-harvest levels, as it is one of the world’s most popular beverages. Common applications include the assessment of coffee ripeness level [91], the identification of pathologies [92,93] and defects [94,95], the evaluation of coffee chemical composition [96,97], and roasting degree [98], and the detection of adulterations [99,100].

In the following sections, the current literature on the authentication of coffee based on species, variety, or geographical origin through MSI and HSI is discussed. In further detail: Section 3.3.1 describes the characteristics of the samples analyzed in the selected works; Section 3.3.2 focuses on the technical aspects of image acquisition and data pretreatment; and Section 3.3.3 examines the multivariate data analysis approaches used for classification and the obtained results.

#### 3.3.1. Sample Characteristics for Coffee Authentication Using HSI and MSI

For ease of reference, the main characteristics of the samples used in the selected works are summarized in Table 4.

**Table 4:** Sample characteristics for the works employing HSI and MSI.

Species	Variety	Bean Condition	Physical Form	Geographical Origin	Ref.
*C. arabica,* *C. canephora* (Robusta)	Not specified	Green	Whole	8 different regions (not specified)	[101]
*C. arabica,* *C. canephora* (Robusta)	Not specified	Green	Whole	Various (not specified)	[102]
*C. arabica,* *C. canephora* (Robusta)	Not specified	Green	Whole	Various (not specified)	[103]
*C. arabica,* *C. canephora* (Robusta)	Not specified	Green, Roasted	Whole	Brazil, Colombia, Costa Rica, Ethiopia, India, Mexico, Honduras, Kenya, Nicaragua, Uganda, Rwanda, Vietnam	[104]
*C. arabica,* *C. canephora* (Robusta)	Not specified	Roasted	Whole	India, Brazil, Peru, Colombia, Mexico, Thailand, Ethiopia, Uganda, Kenya, Vietnam, Indonesia	[105]
*C. arabica*	Not specified	Green	Whole	Alta Mogiana (Minas Gerais, Brazil), Alta Mogiana (São Paulo, Brazil)	[106]
*C. arabica*	Not specified	Green	Whole	Gedio (Ethiopia), Guji (Ethiopia), Maywal (Kenya), Huehuetenango (Guatemala), Kiajibbi (Kenya), Yara (Kenya), West Valley (Costa Rica), Tarrazú (Costa Rica), Santa Maria de Dota (Costa Rica), El Progreso (Honduras), Guatemala (Guatemala), Oaxaca (Mexico), Puebla (Mexico), Estado de Mexico (Mexico), Estelí (Nicaragua), Matagalpa (Nicaragua), Nueva Segovia (Nicaragua), Antioquia (Colombia), Huila (Colombia), Nariño (Colombia), Cusco (Peru), Pasco (Peru).	[107]
*C. arabica,* *C. canephora* (Robusta)	Typica Arabica, Catimor Arabica, Fushan Robusta, Xinglong Robusta	Roasted	Whole	Yunnan and Hainan provinces (China)	[108]

*Coffeea arabica* (*C. Arabica*); *Coffeea canephora* (*C. Canephora*).

As shown in Table 4, the reviewed works display a broad geographical representation, with samples originating from different countries and regions in Central and South America, Africa, and Asia, covering the main coffee production areas. Most studies employed both Arabica and Robusta samples, while the remaining ones used only Arabica samples and focused on the intra-specific classification based on botanical varieties or geographical origin. Only one work [104] explicitly differentiated between traditional and specialty coffee. In this study, the former term refers to commercial samples purchased from a local market, while the latter is commonly used in the coffee industry to indicate coffees scoring above 80 points in the cupping test, a standardized sensorial evaluation performed by trained analysts. Therefore, the application of MSI and HSI to the authentication of specialty coffee, which is characterized by higher quality and economic value, remains largely unexplored.

Green coffee beans were used as samples in the majority of the selected works. This was likely because the roasting process induces numerous chemical reactions, potentially leading to a “masking effect” on some native characteristics that could be species-, variety- or origin-discriminating, thus making the classification task more challenging. Similar considerations may explain why all reviewed studies used whole coffee beans rather than ground samples. In fact, the grinding process, performed post-roasting, could further mask differences relevant to classification. Moreover, when working with ground samples, the main focus would likely be the assessment of their purity degree, i.e., the detection and quantification of adulterations. Since this was one of the exclusion criteria for the present review, studies following this approach were intentionally omitted.

Only three studies employed roasted beans as samples [104,105,108]. In this context, it is important to critically assess the samples’ color—which is related to roasting degree—to ensure that any observed discrimination is indeed due to chemical differences rather than to color variations. On this topic, in Mihailova et al. (2022) [105], the color of the samples was measured in the CIELAB color space and found to vary significantly between Arabica and Robusta. Yet, the outcomes of the study indicated that the most discriminant variables were related to the morphology of the samples, rather than their spectral or color features. Conversely, in Caporaso et al. (2018) [104] and Zhang et al. (2018) [108], color measurement was not performed. Although these works operated in the near-infrared (NIR) region, which is not directly related to color, it is important to consider that some chemical changes triggered by the roasting process, e.g., the formation of melanoidins, can certainly influence NIR spectra. Both studies explicitly addressed this issue: in Zhang et al. (2018) [108], it is specified that all samples were “medium toasted”, while in Caporaso et al. (2018) [104], both Arabica and Robusta samples were roasted applying the same time-temperature combination, resulting in a “medium-high roasting degree”. Overall, it can be concluded that all three studies displayed awareness regarding the importance of accounting for color changes when working on the classification of roasted coffee samples.

#### 3.3.2. Spectral and Spatial Information

Following the same structure as the previous section, the instrumental and technical details regarding the image acquisition and the data pretreatment procedures are listed in Table 5.

As reported in Table 6, most works employed HSI rather than MSI. Since HSI generates a greater amount of data, its prevalence in academic research is to be expected, as it allows for more detailed analyses. However, the increased data volume implies higher computational loads and longer processing times. Consequently, many works employing HSI include the evaluation of the most relevant wavelengths for their specific classification task, which could provide advantageous solutions for the development of faster analytical procedures. One study in particular, Calvini et al. (2017) [103] outlined a complete pipeline encompassing hyperspectral images acquisition, wavelength selection, and the subsequent simulation of a multispectral system.

**Figure 7:**
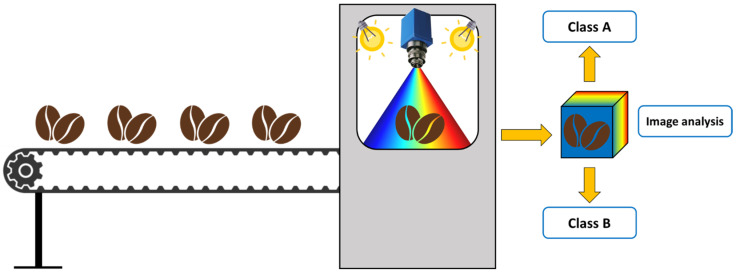
Graphical representation of a HSI-based automated sorting system. Hyperspectral images are acquired while the product moves along the conveyor belt, and then further elaborated to provide the final classification outcome.

The acquisition devices operated primarily in reflectance mode, i.e., they measured the radiation reflected from the sample surface. However, in Gomes et al. (2022) [106], the autofluorescence mode was also used to measure the radiation emitted by the sample fluorophores after photon absorption. The line scan (also referred to as push-broom) configuration was by far the most widely adopted among the reviewed works. In this configuration, the sample is moved along one spatial dimension while the sensor acquires the spectral information across the other, constructing the image one line of pixels at a time. This setup is particularly suitable for applications in industrial settings, as it can be implemented on conveyor belt systems such as the one shown in Figure 7 for continuous online monitoring [109].

The plane scan acquisition configuration, which captures the entire spatial information one spectral channel at a time, was adopted only by the two studies employing MSI [107]. Indeed, this configuration is more typical of MSI, where only a limited number of spectral bands are recorded [109].

All the reviewed studies explored the near-infrared (NIR) region, at least partially. One HSI study [107] worked on a broader spectral range, including both the visible and near-infrared regions (VisNIR), while two MSI studies [105,106] investigated wavelengths belonging to the Vis, NIR, and ultraviolet (UV) spectral ranges. Many spectral preprocessing techniques were tested across the examined works, with Standard Normal Variate (SNV) often emerging as the most effective, either independently or in combination with derivatives. This comes as no surprise, since SNV allows for the correction of light-scattering effects, a common phenomenon in NIR spectroscopy, and to remove physical artifacts affecting the spectral signal, such as those due to the shape of the imaged sample.

Regarding the extraction of relevant features from images, the majority of the studies considered the calculation of average spectra at different levels, i.e., individual beans, groups of beans, or entire images, that were subsequently used for classification purposes. This approach is commonly employed for analyzing image spectral data, and it is certainly useful for reducing the dataset size and the overall computational load while taking into account information related to the average sample composition. However, it should be noted that this approach does not fully exploit the potential of HSI and MSI techniques. Indeed, averaging spectra leads to the loss of valuable spatial information, such as shape, size, and texture, that could improve sample characterization and classification.

Among the research works considered in this review, some studies are found that effectively handle spatial information. For instance, Calvini et al. (2016) [101] proposed the computation of hyperspectrograms as a strategy to reduce the data dimensionality of hyperspectral images while simultaneously retaining the information about spatial variability among pixels. Each HSI image was converted into a signal, named a hyperspectrogram, which is obtained by concatenating the frequency distribution curves of the quantities (i.e., score vectors, Hotelling T2 values, and Q residuals) obtained from PCA. In this manner, hyperspectrograms encode information related to the spatial variability within each image. Two variants of the hyperspectrograms were applied in Calvini et al. (2016) [101], which differ in the way PCA is calculated. In Single Space Hyperspectrograms, a separate PCA model is calculated for each image, while in Common Space Hyperspectrograms, a common PCA model is calculated considering all the images of the dataset together.

In Mihailova et al. (2022) [105], spectral and spatial features were extracted from the images. Considering spatial features, descriptors such as area, length, width, width-to-length ratio, compactness circle, compactness ellipse, among others, were used to characterize the morphology of the beans. These morphological features emerged as the most relevant features for classification, once again highlighting the value of spatial information.

Overall, it appears there is still potential to better exploit the extensive spatial information provided by MSI and HSI techniques.

The importance of spatial-related information has been demonstrated in many studies. For example, Oliveri et al. (2019) [95] employed an object-based calculation of statistical descriptors accounting for spatial information to identify defects in green coffee beans. Considering food products different from coffee, numerous research works reported the integration of spectral information and spatial features to classify samples according to variety or quality attributes [110,111,112]. A similar approach could also be applied to authenticate species, variety, or geographic indication of coffee, making full use of the double spatial/spectral nature of MSI and HIS systems. Of course, this also implies the need to come up with efficient strategies to keep computational demand under control.

#### 3.3.3. Classification Approaches and Metrics for Evaluating and Validation

Table 6 reports the details about model building and classification procedures employed in the selected works.

**Table 6:** Details of the classification procedures for the works employing MSI and HSI. With regard to model performance, more metrics are available in the original articles.

Number of Samples	Classification Task	Level of Image Analysis	Classification Algorithm	Model Optimization	External Validation	Model Performance	Ref.
31 samples (14 Arabica, 17 Robusta).	Classification of green coffee beans based on species and processing method (not relevant to this review)	Image-level	PLS-DA	CV: contiguous blocks with 17 deletion groups. Model dimensionality optimized by minimizing RMSECV	Test set of 83 HSI (corresponding to 7 samples). Models applied to each HSI AS, SSH, and CSH, and data obtained by low-level and mid-level data fusion	NER in prediction of test set: CSH 100.0%; mid-level data fusion (block-scaling) 100.0%	[101]
33 samples (15 Arabica, 18 Robusta).	Classification of green coffee beans based on species	Image-level and pixel-level	- PCA + kNN; - sparse PCA + kNN; - PLS-DA; - sparse PLS-DA	- For (sparse)PCA + kNN: number of PCs and sparsity constraint defined by evaluating efficiency on a fixed monitoring set; - For (sparse)PLS-DA: CV by contiguous blocks with 4 groups. Model dimensionality and number of non-zero variables for each sLV were optimized based on CV efficiency	- For image-level classification: test set of 108 HSI. Models applied to each HSI average spectrum; - For pixel-level classification: test image obtained by merging one HSI of Arabica and one of Robusta of samples belonging to the test set. Models applied to all spectra of the test image	- EFF in prediction of test set (image-level classification): - sparsePCA + kNN 100.0%; - PLS-DA 100.0%; - sparsePLS-DA 100.0%; - EFF in prediction of test image (pixel-level classification): - PCA + kNN 90.6%;	[102]
33 samples (15 Arabica, 18 Robusta).	Classification of green coffee beans based on species	Image-level and pixel-level	- PLS-DA; - sparse PLS-DA	CV by contiguous blocks with 4 deletion groups. Model dimensionality and number of non-zero variables for each sLV optimized by minimizing classification error in CV	- For image-level classification: test set of 108 HSI. Models applied to each image’s average spectrum using: - selected wavelengths (simulation 1); - selected wavelengths plus their differences, ratios, products, and sums (simulation 2); - the most relevant descriptors from simulation 2 (simulation 3); - For pixel-level classification: 2 test images obtained by merging one HSI of Arabica and one of Robusta of samples belonging to the test set. Models applied to all test images’ spectra according to simulations 1, 2, and 3	- EFF in prediction of test set (image-level classification): in range 94.0–100.0% (average 97.45%) across simulations; - EFF in prediction of test images (pixel-level classification): in range 65.1–93.1% (average 80.48%) across simulations	[103]
27 batches (20 Arabica, 7 Robusta).	Classification of green and roasted coffee beans based on species	Object-level (bean-level)	- PCA + LDA; - PCA + QDA (not explicitly mentioned but reported in results table); - SVM	CV with 10 random groups. For SVM, gamma and C values were optimized by grid search	Not clearly described	QDA (% of correct classification): - green coffee: in range 99.80–100.00% depending on pretreatment; - roasted coffee: in range 98.82–100.00%	[104]
35 samples (21 Arabica, 14 Robusta).	Classification of roasted coffee beans based on species	Image-level	OPLS-DA	CV with 7 groups	Test set of 58 MSI. Models applied to each MSI average spectral, morphological, and color features	100% accuracy in the prediction of the test set	[105]
16 samples (10 specialty, 6 traditional).	Classification of green coffee beans into specialty and traditional	Object-level (bean-level)	SVM; Random Forest; XGBoost; CatBoost	CV with 5 groups on 10% of the data (randomly split from the whole dataset)	Test set with 10% of the data (randomly split from the whole dataset). Models applied to reflectance and autofluorescence data of the test set	Accuracy in prediction of test set: SVM: 96%	[106]
24 samples (across 3 continents, 8 countries, 22 regions).	Classification of green coffee beans based on geographical origin at the continental, country, and regional levels	Object-level (each object represents half of the super-pixels of each image)	- PLS-DA; - Linear SVM; - Radial Basis Function kernel SVM; - Random Forest	CV with 10 groups and 10 repeats. For PLS-DA, model dimensionality is optimized using RMSEC and RMSECV. For SVM, gamma and C values were optimized using the highest performance in CV. For RF, maximum depth of each tree, number of trees and number of predictor features per node/split tuned through out-of-bag error rates	Test set with 20% of data. Models applied to the test set spectra (each corresponding to the average of the mean spectra of 25 super-pixels)	Best accuracy values in prediction of test set: - Continental level: 100% SVM with radial basis function - Country level: 100% SVM with radial basis function - Regional level: 96% SVM with radial basis function	[107]
1200 beans (300 per variety)	Classification of roasted coffee beans based on variety	Object-level (bean-level) and pixel-level	SVM	CV performed (not described)	Test set of 8 HSI (corresponding to 2 for each variety). - For object-level classification: models applied to the average spectrum of each bean of the test set; - For pixel-level classification: models applied to all spectra of the test set	- Accuracy in prediction of average spectra by models built on average spectra: 92.25–98.25%; - Accuracy in prediction of individual pixels spectra by models built on random individual pixels spectra (2000 per variety): 77.65–90.89%;	[108]

AS: Average spectrum; Catboost: Gradient boosting with categorical feature support; CSH: Common-space hyperspectrogram; CV: Cross-validation; EFF: Efficiency; kNN: k-nearest neighbors; LDA: Linear discriminant analysis; NER: Non-error rate; OPLS-DA: Orthogonal partial least squares discriminant analysis; PCA: Principal component analysis; PCs: principal components; PLS-DA: Partial least squares discriminant analysis; QDA: Quadratic discriminant analysis; RF: Random forest; RMSEC: Root mean squared error in calibration; RMSECV: Root mean squared error in cross-validation; sLV: Sparse latent variable; SSH: Single-space hyperspectrogram; SVM: Support vector machine; XGboost: Extreme gradient boosting.

As previously mentioned, the sampling process prior to model building is crucial for the development of a robust classification model. The samples should be balanced across classes, while simultaneously providing an adequate representation of the variability within each class. In the selected works, the samples were generally obtained from different batches. To include a broader variability in the classification models, all studies included replicates, either by analyzing different aliquots of each sample, by repeating image acquisition on each aliquot, or by combining both approaches.

All the examined studies followed a discriminant analysis approach to classification. The classification of samples according to their botanical species, i.e., Arabica or Robusta, was the main objective of most works, but other classification tasks, such as the discrimination based on geographical origin [107], variety [108], or between traditional and specialty coffee [106] were also investigated.

Several classification algorithms were tested, among which the most frequent were PLS-DA and SVM. Since the technical details of the classification procedures are beyond the scope of the present review, only a brief description of the two methods is provided. The reader is referred to Ballabio & Consonni (2013) [113] and Brereton & Lloyd (2010) [114] for more information on PLS-DA and SVM, respectively. PLS-DA consists of the application of the PLS regression algorithm to categorical response variables. First, it performs a data dimensionality reduction by identifying latent variables that maximize the covariance between the predictor matrix (**X**) and a binary-coded dummy response matrix representing class membership (**Y**). For each sample, the predicted response values (ŷ), which indicate the likelihood of belonging to each class, are calculated, and the final class assignment is performed accordingly, either by choosing the most probable class or by applying a decision threshold to ŷ. In the selected works, different variants of PLS-DA were tested, which are based on the original framework of the algorithm. For instance, Calvini et al. (2015, 2017) [102,103] employed sparse PLS-DA, which enables feature selection by forcing all model coefficients of irrelevant variables to zero. In Mihailova et al. (2022) [105], orthogonal PLS-DA (OPLS-DA) was used, which removes all variation in **X** not correlated to **Y**, thereby focusing the model on the condition of interest, i.e., the class discrimination [115].

In SVM, the modeled classes are separated by a hyperplane that maximizes the margin between them. If the classes are not linearly separable, non-linear decision boundaries can be introduced using kernel functions. The main idea behind kernel functions is that the data points are mapped into a higher-dimensional feature space where the classification task becomes simpler, ideally allowing for linear separation. Then, the optimal separation boundary is projected back to the original variable space, resulting in a non-linear boundary that better suits the data points distribution.

Another important aspect of classification is the need for model optimization. To this end, all examined works performed a cross-validation step, an iterative resampling process through which the model parameters can be tuned to achieve the best performance while preventing data overfitting. After optimization, the model must be validated on a set of samples that were not used for its own construction. For this purpose, the entire dataset is generally subdivided prior to model building into a training set, which is used for model calibration and cross-validation, and a test set, used for external validation. Alternatively, samples from earlier batches can serve as training data, while subsequent batches are used for external validation. Across the selected works, between 10% and 33% of all samples were used as a test set.

Regarding the classification performances reported in the selected works, the best results were obtained at the image- or object-level, with accuracy and efficiency values ranging roughly from 90% to 100%. These models often made use of average spectra, which allow for mitigating both the heterogeneity in the averaged area and the random noise, thus possibly simplifying the classification task. Calvini et al. (2016) [101] proposed a different strategy for image-level classification, testing hyperspectrograms, which retain both spectral and spatial information. In this study, the classification performance was expressed as non-error rate (NER), which corresponds to the arithmetic mean of the sensitivity values achieved for all the considered classes. The reported NER values ranged from approximately 90% to 100%, and the best performances were obtained when Common Space Hyperspectrograms were used, rather than the average spectra. This reinforces, once again, the high value of the spatial information embedded in each image and its usefulness for classification.

As expected, lower classification performances were recorded for pixel-level classification tasks, where the variability within each hyperspectral or multispectral image is fully exhibited. For example, in Calvini et al. (2015) [102], efficiency values of approximately 80–90% were achieved for the classification of individual pixel spectra. In Zhang et al. (2018) [108], pixel-level classification was attempted through two types of models: the ones built using sample-average spectra and the ones built using a certain number of individual pixel spectra. The sample-average-based models obtained poor performances, with accuracy values of approximately 40%, while the single pixel spectra-based models performed well, with accuracy values in the 80–90% range. These findings allow for one more consideration regarding the evaluation of classification performances. Although models built using average spectra may be sufficient for easier classification tasks, this approach could lead to oversimplistic models. Conversely, models built from individual pixel spectra should be better able to capture the inherent variability within each class. Therefore, depending on the specific task at hand, such models could provide a more robust solution to classification.

In general, the selected works show that MSI and HSI, coupled with multivariate classification methods, are a valid tool for classifying coffee by species, variety, or geographical origin. Still, as discussed above, there is certainly space for more research on this topic. On one hand, the analytical techniques could be more thoroughly exploited, especially with regard to spatial information. On the other hand, classification tasks such as those addressed in Gomes et al. (2022) [106], which focused on discrimination between traditional and specialty coffee, are still largely underexplored.

## 4. Conclusions

Digital image analysis and spectral imaging jointly demonstrate significant promise for the botanical and geographical classification of coffee, offering rapid, non-destructive, and increasingly accessible alternatives to conventional analytical methods.

In digital image-based approaches, high classification performance has been reported for species and geographical authentication studies. These investigations relied solely on color descriptors, exploring different color spaces such as RGB, HSV, and grayscale representations. Authentication tasks were exclusively addressed using the one-class classifier DD-SIMCA, demonstrating its strong capability for target-class recognition. However, insufficient control of roast degree in these studies introduces a potential confounding factor, as roasting-induced color variations may overlap with botanical or geographical differences, potentially limiting the generalizability of the reported results.

In contrast, the classification of different coffee varieties was performed using discriminant methods, for which a broader range of image features was explored. At the varietal level, in addition to color variables, texture parameters derived from co-occurrence matrices, including entropy, correlation, homogeneity, and contrast, as well as shape descriptors, were employed. It is worth mentioning that shape and texture parameters are currently underexplored in coffee classification, and their adoption remains a possible key for harder tasks such as discrimination of the origin of the same varieties. In these studies, non-linear discriminant techniques were predominantly used, with SVM being the most widely adopted approach and consistently yielding high classification performance. This superior image classification by non-linear models may relate to their hierarchical and high-dimensional nature, which requires complex non-linear relations.

Furthermore, the application of end-to-end convolutional neural networks expanded the discriminative capacity of image-based classifiers. However, this improvement comes with an inherent trade-off between predictive performance and interpretability, as the learned features encode complex visual patterns that are not directly attributable to specific physical or chemical characteristics. In addition, several studies lacked external validation procedures and, in some cases, did not clearly report sampling size or experimental design, limiting the assessment of model robustness and reproducibility.

Regarding spectral imaging, the selected studies reported satisfactory classification performance, supporting its effectiveness for coffee authentication. Most works focused on discrimination by species, whereas cultivar identification, geographical origin discrimination, and differentiation between common and specialty coffee remain comparatively underexplored and represent promising research directions. The majority of studies were conducted on whole green coffee beans to avoid the masking effects of roasting. However, extending research to roasted and ground coffee could enhance applicability in later stages of the supply chain and strengthen protection against fraud.

The spatial dimension of HSI and MSI remains largely underutilized. Many studies relied on average spectra for classification purposes, effectively neglecting spatial information. Although this approach simplifies data handling and reduces computational complexity, it discards potentially valuable features related to size, shape, texture, and spatial distribution. As an alternative, several studies emphasized dimensionality reduction strategies, reporting good results by approaching the selection of informative wavelengths, hyperspectrogram compression, and data-fusion approaches.

All HIS and MSI studies adopted a discriminant analysis approach, primarily modeling inter-class differences rather than intra-class variability. A broad range of chemometric algorithms was evaluated, including both linear and non-linear classifiers, with PLS-DA and SVM being the most frequently applied. Classification was performed at the pixel, object, and image levels, with higher performances generally observed at the object and image levels, where spectral averaging mitigates heterogeneity and noise. In contrast, pixel-level approaches better capture the intrinsic sample variability and may provide a more realistic assessment of model robustness. Reinforcing that, greater exploitation of spatial information provided by MSI and HSI could therefore further strengthen coffee authentication strategies.

Collectively, the reviewed evidence supports these imaging approaches as viable foundations for rapid and non-destructive coffee authentication. Continued methodological refinement and future research in integration into automated platforms will make their transition from experimental applications to industrial quality-control systems possible.

## Figures and Tables

**Table 1:** Physical and biological parameters of coffee samples used in the digital imaging studies.

Species	Variety	Bean Condition	Physical Form	Geographical Origin	Equipment	Ref.
*C. arabica*	Harari, Khawlani, Nabari, Laqamt, and Bariah	Roasted and green	Whole	Saudi Arabia	Smartphone iPhone 14	[33]
*C. canephora* (Robusta)	Amazonian Robusta	Roasted	Ground	Rondonia, Brazil	Smartphone Redmi Note 11	[17]
*-C. canephora* (Robusta) -*C. arabica*	Not specified	Roasted	Powdered (instant coffee)	-South Bahia and Espirito Santo, Brazil -undeclared provenance	Scanner HP Deskjet 2540	[16]
*-C. arabica* *-C. canephora* (Robusta) *-C. liberica*	Not specified Robusta Excelsa	Roasted and green	Whole	Not specified	Not specified	[34]
*C. arabica*	Harrar, Jimma, Limu, Sidama, Wellega	Green	Whole	Ethiopia	Not specified	[35]
*-C. arabica* *-C. canephora* (Robusta) *-C. liberica*	Not specified Robusta Excelsa	Roasted	Whole	Indonesia	Smartphone (Sony a6000)	[15]
*-C. canephora* (Robusta) *-C. arabica*	Not specified	Green	Whole	Indonesia	Andromax A mobile camera	[7]
*C. arabica*	Logan and regular	Green	Whole	Indonesia	Nikon Coolpix A10 camera	[36]

**Table 2:** Parameters and features extracted from the coffee beans in digital image studies.

Resolution and the Selected Area (pixel)	Illumination Setup	Shape Features	Color Coordinate	Color and Texture Features	Image Pre-Treatment	Raw Image Processing	Ref.
12 MP 265 × 265	LED lightbox and sunlight	None	Grayscale	RGB features automatically extracted by CNN	Crop and flip	Grayscale conversion, background removal, binarization, hole filling, noise removal (bwareaopen)	[33]
50 MP 8165 × 6124	Studio black box with white LED strips (6500 K)	None	RGB	Average RGB histograms	None	Select ROI and acquisition of the RGB histogram	[17]
600 dpi 250 × 250	Not Specified	None	Grayscale, RGB, HSV	Grayscale, RGB, HSV, and (Grayscale + RGB + HSV)	Crop	Select ROI and acquisition of the RGB histogram	[16]
N.S. 150 × 150	Not Specified	Shape features automatically extracted by CNN	RGB	RGB features automatically extracted by CNN	Not Specified	Not Specified	[34]
12 MP + 5 MP Dual rear camera 360 × 360	Not Specified	Shape features automatically extracted by CNN, HOG, and CNN + HOG	RGB and Grayscale	Color features automatically extracted by CNN, HOG, and CNN + HOG	Thresholding and K-means algorithms for image segmentation.	Image resizing, filtering, contrast enhancement, noise removal, grayscale conversion, and segmentation	[35]
12 MP 640 × 480	Studio minibox with LED	Area, perimeter, length, width, equivalent diameter, aspect ratio, ratio of the equivalent ellipse axis, eccentricity, compactness, the Heywood circularity factor, extent, solidity, and arc	Grayscale	none	Resized the original images from 6000 × 3376 to 640 × 480 pixels	Select ROI and gray-scaling, and histogram equalization of images, and “Gain ratio”	[15]
3.5 MP 1000 × 1000	Not Specified	None	Grayscale	Energy, Correlation, Homogeneity, Contrast	Crop	Grayscale	[7]
16 MP 1343 × 1349	Consistent lighting conditions via constant fluorescent	None	Gray, HSL, HSV, and L*a*b*	Blue_Mean, Hue_Entropy, Gray_Inverse, S_HSL_Correlation, and Green_Cluster.	Crop	None	[36]

Not specified; (N.S.); Red Green Blue color coordinate (RGB); Megapixel (MP); Light-Emitting Diode (LED); Hue, Saturation, Value (HSV); Hue, Saturation, Lightness (HSL); CIELAB color space (L*a*b*); Convolutional Neural Networks (CNN); Histogram of Oriented Gradients (HOG); Region of Interest (ROI).

**Table 3:** Sample set and performance parameters of the digital image models.

Number of Samples/Images	Classification Task	Classification Algorithm	Cross-Validation	Model Optimization	External Validation	Model Performance	Ref.
N.S./12,310 (four different roast levels of five *C. arabica* varieties: Harari: 2529, Bariah: 2902, Khowlani: 2346, Legamti: 2015, Nabari: 2518)	Classify the 5 varieties among 4 roasting levels	CNN (SqueezeNet) and Vision Transformer enhancement	K-fold CV with k = 6, maximization of model’s accuracy, recall, precision, and F1 scores	A validation dataset composed of 20% of the images for hyperparameter training, minimizing validation loss	A test set validation of 10% of the dataset images	Overall accuracy 78%; 78% recall, 77% precision, 76% F1 score	[33]
300 samples (100 indigenous Amazonian *C. canephora*, 100 *C. canephora,* conilon, and 100 adulterated coffees (one image per sample)	Authentication of indigenous *C. canephora* against *C. canephora*, conilon and adulterated indigenous coffee	DD-SIMCA	Procrustes cross-validation and optimizing the sensitivity of cross-validation and test models	-	Prediction of the 200 external samples (100 *C. canephora*, conilon, 100 adulterated indigenous *C. canephora*)	Sensitivity: 98%; Specificity against *C*. *canephora*, conilon: 95%; Specificity against adulterated indigenous *C. canephora:* 95%	[17]
90 samples (30 *C. canephora* from Southern Bahia, 30 *C. canephora* from Espirito Santo, 30 coffees of undeclared provenance) /3 images per sample	Authentication of Southern Bahia *C. canephora* against Espirito Santo *C. canephora*/discrimination between Espirito Santo and undeclared provenance samples	DD-SIMCA and PLS-DA	for DD-SIMCA: maximization of TP rate/for PLS-DA: LOO-CV and minimization of RMSECV	-	Test set composed of 33% of the dataset selected by Kennard Stone algorithm	For DD-SIMCA, 100% sensitivity; 100% efficiency/for PLS-DA, 100% accuracy	[16]
N.S./200 images (four species, 50 images per class)	Classification of four different coffee species	CNN (basic architecture)	10-fold CV with 20% of the data in the deletion groups	Adam optimizer used to minimize the loss function	No external validation informed	Overall: 84% accuracy 86% precision, 84.1% recall and 84.1% F1-Score	[34]
N.S./600 images augmented to 1200. From five *C. arabica* varieties (240 of each): Harar, Jimma, Limu, Sidama, Wellega	Classification of the 5 varieties	Deep learning models for feature extraction and SVM	Not clearly described	Not clearly described	A test set of 20% of the dataset was separated	SVM: 97.5% accuracy	[35]
N.S./400 images in total. From four species (Arabica, Excelsa, Liberica, Robusta), 100 images of each	Discrimination between the four coffee species	Naïve Bayes, ANN, SVM, C.45 and decision tree	10-fold CV	Not clearly described	No external validation informed	For cross-validation set: decision tree; Precision: 99.5%; Recall 99.5%; Accuracy 99.5%	[15]
N.S./58 images (29 from *C. arabica* and 29 from *C. canephora*)	Discrimination between *C. arabica* and *C. canephora* coffees	SVM, Decision tree, Logistic Regression, Naive Bayes	10-fold CV	Not clearly described	No external validation informed	For the cross-validation: SVM F1: 98.3%; Precision: 98.3%; Recall: 98.3%	[7]
N.S./528 images among 4 purity levels	Discriminate purity levels of Palm Civet Coffee	K-NN, Random forest, and SVM	5-fold CV for K-NN	Manually assaying several parameters to find the optimal classification accuracy	The test set composed of randomly selected 30% of the data.	Using a Random Forest with Grey Wolf Optimizer: Accuracy 98.10%	[36]

CCN: Convolutional neural networks; SVM: Support vector machines; LOO-CV: leave-one-out cross-validation; RMSECV: Root Mean Square Error of Cross-Validation; K-NN: K-nearest neighbors; DD-SIMCA: Data-driven soft independent modelling of class analogy; ANN: artificial neural network; PLS-DA: Partial least squares discriminant analysis.

**Table 5:** Details of the image acquisition and data pretreatment for the works employing MSI and HSI.

Number of Images	Instrumental Technique	Illumination Setup	Spatial Resolution (pixels)	Spectral Range (nm)	Number of Wavelengths	Extracted/Selected Features	Raw Data Processing	Data Pretreatment	Ref.
370	HSI line scan	Integrating cylinder + halogen lamps	320 × 256	955–1700	150	- Average spectrum (AS) of each HSI; - Single-space hyperspectrogram (SSH) and common-space hyperspectrogram (CSH) of each HSI; - Low-level data fusion (in-sequence merging of AS, SSH and CSH blocks); - Mid-level data fusion (in-sequence merging of scores obtained by PLS-DA on AS, SSH and CSH blocks)	Conversion into reflectance values, internal calibration to reduce variability over time, and background removal	- For AS: SNV + mean centering; - For SSH and CSH: mean centering; - For data combined by low-level data fusion: each block pretreated separately as described above, then block-scaling; - For data combined by mid-level data fusion: block-scaling or autoscaling	[101]
396	HSI line scan	Not specified	320 × 256	955–1700	150	- Average spectrum of each HSI; - Selection of most relevant variables through sparse methods	Conversion into reflectance values, internal calibration to reduce variability over time, and background removal	SNV + first derivative + mean centering	[102]
396	HSI line scan	Not specified	320 × 256	955–1700	150, then MSI simulation with 4 selected wavelengths: 1150 nm, 1200 nm, 1250 nm, 1400 nm	- Average spectrum of each HSI; - 50 random spectra for each HSI of training set; - Optimal wavelengths. - Reflectance values at selected wavelengths; - Descriptors derived from reflectance values (squared value of each channel, differences, ratios, products and sums between pairs of channels)	Conversion into reflectance values, internal calibration to reduce variability over time, and background removal	Autoscaling	[103]
Not specified	HSI line scan	Two 500 W incandescent lamps	320 × 256	980–2500	256	- Unweighted average absorbance spectrum of each bean	Bad pixels and spikes removal, segmentation to select each bean	Several pretreatments tested: SNV, first and second derivative, MSC, detrending, normalization	[104]
175	MSI plane scan	Strobed LEDs at 19 wavelengths	2992 × 2992	365–970	19 wavelengths: 365, 405, 430, 450, 470, 490, 515, 540, 570, 590, 630, 645, 660, 690, 780, 850, 880, 940, 970 nm	- Spectra of a 5 × 4 mm ROI within each bean; - Morphological features of each bean: area, length, width, width-to-length ratio, compactness circle, compactness ellipse, beta shape a, beta shape b; - Color features of each bean: CIELAB coordinates (L*, a*, b*), saturation, hue; - Average spectral, morphological and color features of each image - Most important variables	- Background removal and segmentation to select each bean; - Transformation of each image using normalized canonical discriminant analysis (nCDA)	SNV + autoscaling	[105]
Not specified	MSI plane scan	Strobed LEDs at 19 wavelengths	2192 × 2192	- 365–970 nm (reflectance mode); - 365–660 nm (autofluorescence mode)	- Reflectance mode: 19 wavelengths (365, 405, 430, 450, 470, 490, 515, 540, 570, 590, 630, 645, 660, 690, 780, 850, 880, 940, 970 nm). - Autofluorescence mode: 28 excitation/emission combination (13 wavelengths: 365, 405, 430, 450, 470, 490, 515, 540, 570, 590, 630, 645, 660 nm; and 4 long-pass filters: 400, 500, 600, 700 nm)	- Reflectance and autofluorescence data for each bean; - Importance of each variable for the classification task	- Radiometric and geometric calibration, background removal, and segmentation to select each bean; - Transformation of each image using normalized canonical discriminant analysis (nCDA)	Autoscaling	[106]
72	HSI line scan	150 W tungsten halogen lamp	320 for each line	700–1700	235 before removing wavelengths from 550 nm to 700 nm	- Average spectrum of each super-pixel (50 per image), then averaged in halves (resulting in 2 spectra per image); - Discriminant wavelengths	Conversion into reflectance values, background removal, division of each image into 50 super-pixels	Several row-wise pretreatments tested (MSC, SNV, first and second derivative, smoothing) + mean centering	[107]
24	HSI line scan	Two 150 W tungsten halogen lamps	320 × 256	874–1734 nm	172	- Average spectrum of each sample; - 2000 random spectra per variety (selected from training set images); - Optimal wavelengths	Conversion into reflectance values and background removal	- Median filters (size 3 × 3, 7 × 7, 11 × 11, 15 × 15) on grayscale images at every wavelength; - Several pretreatments tested on spectra: moving average (window size 7, 11, 15, 19, 23), wavelet transform and empirical mode; - Second derivative of cultivar-average spectra to select optimal wavelengths	[108]

AS: Average spectrum; CIELAB: Color space L* a* b*; CSH: Common-space hyperspectrogram; LED: Light-emitting diode; MSC: Multiplicative scatter correction; nCDA: normalized canonical discriminant analysis; ROI: Region of interest; SNV: Standard normal variate; SSH: Single-space hyperspectrogram; PLS-DA: Partial least squares discriminant analysis.

## Data Availability

No new data were created or analyzed in this study. Data sharing is not applicable to this article.

## Supplementary Materials

The following supporting information can be downloaded at: https://www.mdpi.com/article/10.3390/foods15050821/s1, File S1: PRISMA Checklist.
